# The significance of the site of origin in primary peritoneal carcinosarcoma: case report and literature review

**DOI:** 10.3332/ecancer.2013.295

**Published:** 2013-03-07

**Authors:** Anupama Rajanbabu, Sheikh Zahoor Ahmad, Vijaykumar D K, Pavithran K, Santhosh Kuriakose

**Affiliations:** 1 Department of Surgical and Gynecologic Oncology, Amrita Institute of Medical Sciences, Amrita Vishwavidyapeetham, India; 2 Department of Surgical Oncology, Florence hospital, Srinagar, Kashmir, India; 3 Department of Surgical and Gynecologic Oncology, Amrita Institute of Medical Sciences, Amrita Vishwavidyapeetham, India; 4 Department of Medical Oncology, Amrita Institute of Medical Sciences, Amrita Vishwavidyapeetham, India; 5 Department of Gynecology, Govt. Medical College, Calicut, India

**Keywords:** primary peritoneal carcinosarcoma, malignant mixed Mullerian tumour, extrauterine carcinosarcoma

## Abstract

Primary peritoneal carcinomas are rare, highly aggressive malignant neoplasms containing both sarcomatous and carcinomatous elements. Surgical debulking is the mainstay of treatment for primary peritoneal carcinomas. Systemic chemotherapy is advised in all cases because of the early spreading of these tumours. We report on a case of primary peritoneal carcinosarcoma occurring in a 22-year-old woman.

Carcinosarcomas are highly aggressive biphasic neoplasms composed of carcinomatous and sarcomatous elements. Mostly occurring in the female genital tract in elderly postmenopausal women, they have also been described in head and neck, gastrointestinal tract, biliary tract and peritoneum [1]. Primary peritoneal carcinosarcomas are extremely rare with the majority of the tumours occurring in the pelvic peritoneum, followed by decreasing frequency on the serosal surface of the colon, retroperitoneum, anterolateral abdominal peritoneum, and omentum [2]. They have poor outcomes despite being managed with upfront surgery and chemotherapy [3]. After the first case described by Ober and Black [4] in 1955, only 35 cases have been reported in the English literature. So far, all the reported cases are in females over the age of 40. We report a case of primary peritoneal carcinosarcoma occurring in a 22-year-old woman.

## Case history

A 22-year-old unmarried female presented with a two month history of non-colicky lower abdominal pain. Clinically, there was a 10 × 12 cm mobile mass arising from the pelvis reaching up to the umbilicus. Contrast enhanced computerised Tomography (CECT) abdomen revealed an 11 × 11 × 9 cm heterogeneously enhancing predominantly cystic mass in the lower abdomen superior to the bladder and uterus extending upwards to aortic bifurcation. It was abutting the anterior surface of the left psoas muscle and left external iliac artery, and anteriorly reaching up to the parietal wall. The ovaries, uterus, and rest of the viscera were normal. Serum tumour markers (CA-125, AFP, and ß-HCG) were within normal limits. The patient underwent an exploratory laparotomy. Intra-operatively minimal hemorrhagic ascites were noted. A 12 × 10 cm heterogeneous solid cystic mass was noted in the sigmoid mesentery adherent to the psoas fascia posteriorly. Both ovaries and uterus were seen to be normal. The mass was excised completely with a segment of sigmoid colon.

**Figure 1:**
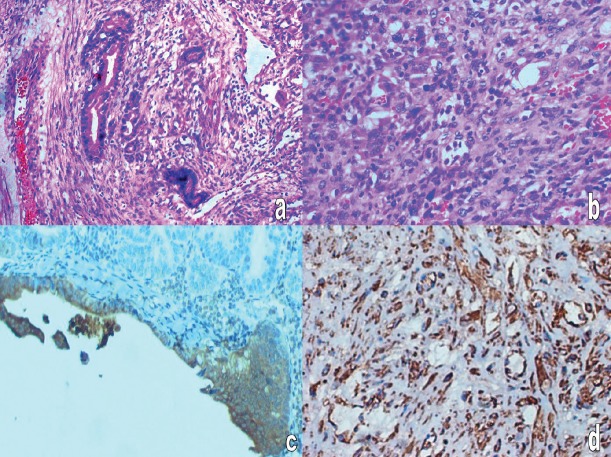
Histopathology: (a) adenocarcinomatous area -H&E-400 X, (b) sarcomatous area with giant cells and atypical mitosis H&E-400 X, (c) cytokeratin positivity in carcinomatous area, (d) vimentin positivity in sarcomatous area.

Gross pathology showed an intact 13 × 9 × 10 cm mass with a segment of sigmoid colon. Cut section showed grey white fleshy solid areas, interspersed areas of haemorrhage, along with multiple cystic spaces filled with thick fluid. Microscopic examination showed that the cystic areas were lined by tall columnar cells arranged in papillary and cribriform pattern and with pale oeosinophilic cytoplasm and pleomorphic nuclei. Solid areas showed sheets of spindly cells with moderate cytoplasm and elongated pleomorphic nuclei. There were five to six mitosis/10 HPF. Neoplasm was adherent to the serosa of the sigmoid colon. Adjacent colonic mucosa was normal. The epithelial cells were positive for cytokeratin (CK) and focally for carcinoembryonic antigen (CEA) and the spindly cells were positive for vimentin (Figure 1).

Post operatively she was given six cycles of chemotherapy with ifosfamaide and cisplatin. CT scan taken after completion of chemotherapy showed a recurrent lesion within the abdomen measuring 6 × 5.8 × 4.3 cm size. In view of the progressive disease, carboplatin, and paclitaxel combination chemotherapy was started. CT scan taken after two cycles showed that the disease was progressing on treatment and hence, a cycle of Adriamycin with cyclophosphomide was given. There was no clinical response with the third line chemotherapy also and the patient expired 9 months after starting the treatment.

## Discussion

Carcinosarcomas or malignant mixed Mullerian tumours are rare, highly aggressive biphasic neoplasms comprised of carcinomatous and sarcomatous components, which accounts for one to three per cent of all uterine malignancies [5] and even more rarely are described to occur in head and neck, gastrointestinal tract, biliary tract and peritoneum [1]. The carcinosarcomas at extragenital sites have been postulated to arise from pre-existing foci of endometriosis, Mullerian duct remnants, or secondary Mullerian system; all of which are derivatives of coelomic epithelium [6]. The histopathological features of this tumour are identical to uterine carcinosarcomas. Uterine carcinomas are now classified as high-grade carcinomas since *in vitro *studies, immunohistochemistry, and molecular studies have shown that they are derived from monoclonal cancer cells which exhibit sarcomatous metaplasia [7] and the behaviour of these tumours is determined by the epithelial component.

Literature search revealed 35 cases of primary peritoneal carcinosarcomas [8, 12–15]. The site of origin varied from pelvic and cul-de-sac peritoneum, uterine serosa, colonic or rectal serosa to abdominal wall peritoneum or retroperitoneum and omentum. We classified the patients into three groups based on the site of origin of tumour; Group 1 arising from pelvic peritoneum/uterine serosa, Group 2 arising from the colonic/rectal peritoneum, Group 3 arising from other peritoneal surfaces. Fourteen patients had tumour arising from the pelvic peritoneum/uterine serosa (Table 1), 11 patients had tumour arising from colonic/rectal peritoneum (Table 2) and 11 patients had tumour arising from omentum, retroperitoneum or abdominal wall peritoneum (Table 3).

Owing to the rarity of primary peritoneal carcinosarcomas, limited data regarding the management exists. The mainstay of treatment is surgical debulking, but most cases of carcinosarcomas have wide spread metastasis at the time of presentation, making optimal tumour debulking difficult [8]. Systemic chemotherapy is advised in all cases irrespective of stage because of early tumour spread. Platinum in combination with ifosfamide were the preferred agents [9]. However, if the tumour behaviour is determined by the epithelial component, data regarding chemotherapy can be extrapolated from the treatment for ovarian cancers. Platinum and taxane combination chemotherapy has given more than 2 years median survival in patients with ovarian carcinosarcoma [10, 11]. It seems logical that these should be used as first line agents for patients with primary peritoneal carcinosarcomas also. There is not enough data to support the role of radiotherapy in extragenital carcinosarcoma [12].

**Figure 2:**
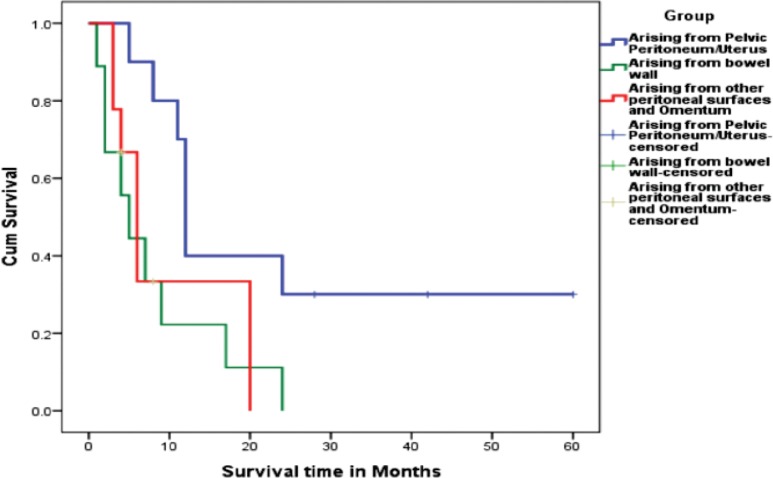
Kaplan Meir survival graph showing the difference in survival between the three groups.

Literature review shows that all patients were managed by surgery followed by chemotherapy when possible (Tables 1–3). Overall survival of all the three groups was compared. Patients belonging to Group 1, who had disease arising mostly from pelvic peritoneum and cul-de-sac, were found to have an average survival of 21.5 months. Group 2 patients with tumour arising from colonic/rectal peritoneum had an average survival of 7.6 months, and the third group had an average survival of only 4.3 months. Log rank analysis (Mantel–Cox) showed that survival time of Group 2 and Group 3 are significantly different from Group 1 (*p *= 0.016 and *p *= 0.040). There is no significant difference in survival between Group 2 and Group 3 (*p *= 0.696). This indicates that a patient with primary peritoneal carcinosarcoma with tumour originating from pelvic peritoneum or uterine serosa is having better survival than a patient with tumour originating from other peritoneal surface. The Kaplan–Meir survival graph is given in Figure 2.

## Conclusion

Primary peritoneal carcinosarcomas are rare tumours associated with a poor prognosis. Tumours arising from the bowel serosa, abdominal wall, omentum or retroperitoneum seem to carry a poorer prognosis than those arising from the pelvic peritoneum. Since these tumours are similar to uterine carcinosarcomas where recent evidence has shown that the tumour behaviour is dictated by the epithelial component, chemotherapy with platinum taxane combination may work better as first line therapy.

## Figures and Tables

**Table 1:** Primary peritoneal carcinosarcomas arising from the pelvic peritoneum/uterine serosa.

No.	Author (year of publication)	Age	Site of origin	Treatment	Follow-up duration (months)	Status at last follow-up
1	Ober and Black (1955)	74	Pelvic peritoneum	Surgery, RT	5	Expired
2	Marchevsky *et al* (1980)	40	Cul-de-sac peritoneum	Surgery, CT (adriamycin, cisplatin)	12	Expired
3	Campins *et al* (1986)	58	Pelvic peritoneum	Surgery	?	?
4	Chen and Wolk (1987)	52	Pelvic peritoneum	Surgery, RT	11	Expired
5	Solis *et al* (1991)	54	Cul-de-sac peritoneum	Surgery	?	?
6	Garamvoelgyi *et al* (1994)	59	Pelvic peritoneum	Surgery, CT (cisplatin, doxorubicin, ifosfamide)	24	Expired
7	Garamvoelgyi *et al* (1994)	64	Cul-de-sac peritoneum	Surgery, RT, CT (ifosfamide)	8	Expired
8	Garamvoelgyi *et al* (1994)	84	Uterine subserosa	Surgery	12	Expired
9	Mira *et al* (1995)	62	Pelvic peritoneum	Surgery	28	Alive
10	Rose *et al* (1997)	57	Cul-de-sac and uterine serosa	Surgery, CT (cisplatin, ifosfamide)	42	Alive
11	Sumathi *et al* (2002)	77	Pelvic peritoneum	Surgery	Nil	Expired
12	Dincer *et al* (2002)	50	Pelvic peritoneum	Surgery, CT (anthracyclin), Anti-angiogenesis agents	?	?
13	Ko *et al* (2005)	45	Pelvic peritoneum, cul-de-sac	Surgery, RT CT (cisplatin, ifosfomide)	60	Alive
14	Kurshumliu *et al* (2011)	72	Pelvic peritoneum	Surgery, CT (carboplatin)	12	Expired

**Table 2.** Primary peritoneal carcinosarcomas arising from the colonic/rectal peritoneum.

No.	Author (year of publication)	Age	Site of origin	Treatment	Follow-up duration	Status at last follow-up
1	Weiss-Carrington (1977)	77	Caecal peritoneum	Surgery	1 week	Expired (Pulmonary embolism)
2	Chumas *et al* (1986)	67	Rectal peritoneum	Surgery, palliative chemotherapy	24 months	Expired
3	El-Jabbour (1989)	76	Ascending colon peritoneum	Surgery	2 weeks	Expired
4	Ohno *et al* (1989)	66	Descending and sigmoid colon	Surgery, CT (cyclophoshomide)	17 months	Expired
5	Fenoglio-Preiser *et al* (1990)	?	Caecal peritoneum	Surgery	?	?
6	Nimaroff *et al* (1993)	82	Sigmoid colon peritoneum	Surgery, CT ( cisplatin, adraimycin, ifosfamide)	5 months	Expired
7	Choong *et al* (1994)	63	Serosa of the sigmoid colon	Surgery	?	?
8	Mira *et al* (1995)	83	Cecal peritoneum	Surgery	7 months	Expired
9	Naniwadekar *et al* (2009)	76	Rectosigmoid peritoneum	Surgery , CT (ifosfomide , cisplatin)	<2 months	Expired (neutropenia)
10	Kanis *et al* (2011)	80	Rectosigmoid peritoneum	Surgery, CT (platinum, taxane, gemcitabine)	4 months	Expired
11	Our case (2013)	22	Sigmoid peritoneum	Surgery, CT (ifosfomaide cisplatin)	9 months	Expired

**Table 3.** Primary peritoneal carcinosarcomas arising from other peritoneal surfaces.

No.	Author (year of publication)	Age	Site of origin	Treatment	Follow-up duration	Status at last follow-up
1	Ferrie and Ross (1967)	47	Abdominal retroperitoneum	Surgery	?	?
2	Hermann and Tessler (1983)	72	Abdominal posterior peritoneum	Surgery, CT (adriamycin, cytoxan, DTIC, vincristin)	6 months	Expired
3	Hasiuk *et al* (1984)	77	Abdominal posterior peritoneum	Biopsy only	20 days	Expired
4	Garde *et al* (1991)	65	Diaphragm peritoneum	Surgery, CT (cisplatin, adriamycin, ifosfamide)	4 months	Expired
5	Rose *et al* (1997)	71	Peritoneum of liver and other surfaces	Surgery, CT ( cisplatin, ifosfamide)	6 months	Expired
6	Rose *et al* (1997)	67	Omentum and peritoneum	Surgery, CT (cisplatin, ifosfamide)	3 months	Expired
7	Shintaku and Masumoto (2001)	51	Retroperitoneum, lateral pelvic wall	Surgery, CT (epirubicin, carboplatin)	4 months	Alive
8	Sumathi *et al* (2002)	87	Omentum, pelvic peritoneum	Surgery	?	?
9	Wei *et al* (2002)	67	Omentum	Surgery, CT (cisplatin, pirarubicin, etoposide)	8 months	Alive
10	Hussein *et al* (2009)	65	Peritoneum	Biopsy only	3 months	Expired
11	Kanis *et al* (2011)	57	Omentum	Surgery, CT (platinum, taxane, gemcitabine)	4 months	Alive
